# A Year in Hypoxia: Epibenthic Community Responses to Severe Oxygen Deficit at a Subsea Observatory in a Coastal Inlet

**DOI:** 10.1371/journal.pone.0045626

**Published:** 2012-09-19

**Authors:** Marjolaine Matabos, Verena Tunnicliffe, S. Kim Juniper, Courtney Dean

**Affiliations:** 1 School of Earth and Ocean Sciences, University of Victoria, Victoria, BC, Canada; 2 NEPTUNE Canada, University of Victoria, Victoria, BC, Canada; 3 VENUS, University of Victoria, Victoria, BC, Canada; University of Southampton, United Kingdom

## Abstract

Changes in ocean ventilation driven by climate change result in loss of oxygen in the open ocean that, in turn, affects coastal areas in upwelling zones such as the northeast Pacific. Saanich Inlet, on the west coast of Canada, is a natural seasonally hypoxic fjord where certain continental shelf species occur in extreme hypoxia. One study site on the VENUS cabled subsea network is located in the hypoxic zone at 104 m depth. Photographs of the same 5 m^2^ area were taken with a remotely-controlled still camera every 2/3 days between October 6^th^ 2009 and October 18^th^ 2010 and examined for community composition, species behaviour and microbial mat features. Instruments located on a near-by platform provided high-resolution measurements of environmental variables. We applied multivariate ordination methods and a principal coordinate analysis of neighbour matrices to determine temporal structures in our dataset. Responses to seasonal hypoxia (0.1–1.27 ml/l) and its high variability on short time-scale (hours) varied among species, and their life stages. During extreme hypoxia, microbial mats developed then disappeared as a hippolytid shrimp, *Spirontocaris sica*, appeared in high densities (200 m^−2^) despite oxygen below 0.2 ml/l. The slender sole *Lyopsetta exilis* was abundant in severe hypoxia and diminished as oxygen increased in the summer. This planktivore may be responding to changes in the depth of the diurnal migration of zooplankton. While the squat lobster *Munida quadrispina* was common at all times, juveniles disappeared in fluctuating conditions. Despite low oxygen conditions, animal densities were high indicating that the risk from hypoxia is balanced by factors such as food availability and escape from less tolerant predators. As hypoxia increases on the continental shelf, we expect benthic communities to become dominated by low diversity, hypoxia-tolerant species of low commercial significance.

## Introduction

Dissolved oxygen concentration is a variable of fundamental biological importance in the oceans [1]. Recent decades have seen accumulating evidence – and growing concern - that low oxygen or ‘hypoxic’ conditions are spreading in marine coastal ecosystems throughout the world [2]. Two phenomena are implicated: 1) eutrophication caused by increasingly intensive agricultural practices, industrial activities, and population growth [1], [3], and 2) climate change that has increased regional upwelling events and sea surface temperatures, leading to shifts in wind patterns, reduced oxygen solubility, and greater water stratification [4]–[7]. While hypoxia (<1.4 ml/l; [1]) naturally occurs in oxygen minimum zones, deep basins, upwelling systems and fjords, long term studies have noted an increase in the number of hypoxic zones as well as their severity, extent and duration [5], [8]–[10].

Oxygen is the key terminal electron acceptor in aerobic energy metabolism in all living organisms. Marine hypoxia has adverse effects on growth, survival, reproduction, recruitment and behavior [2], [11]–[14]. Low oxygen incursions in coastal areas can cause mass mortality [5], shifts in species distributions, reduced biodiversity [15], and habitat loss for commercially important species [16]. Where hypoxia develops seasonally such as in coastal embayments and upwelling regions, the effects on benthic communities mostly depend on the severity and duration of hypoxia. Oxygen tolerances and thresholds vary among organisms [17], [18] with some showing marked tolerance to life in low oxygen concentration environments [19]–[21].

**Figure 1:**
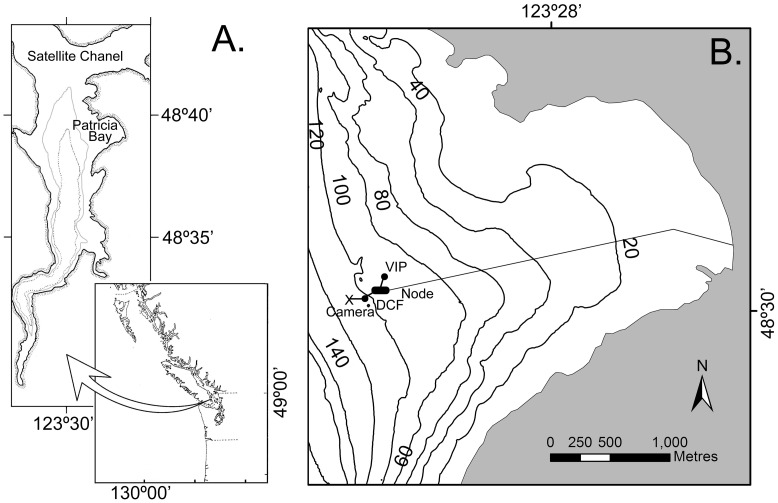
Study site. A. Saanich Inlet is on southern Vancouver Island, British Columbia on the Canadian Pacific coast. B. In Patricia Bay the *Camera,* located on a muddy bottom, was connected to a seafloor platform (*DCF*) that held the power controller and a CTD and O_2_ sensor; the *Cable* connects the VENUS Saanich *Node* to the Shore Station. The Node also serves the nearby instrument platform (*VIP*).

Saanich Inlet, at the southern end of Vancouver Island, Canada, is a naturally hypoxic basin (Figure 1). A shallow sill (70 m) at the mouth isolates the deep basin (215 m) that experiences seasonal deep-water anoxia as a result of high primary productivity and subsequent degradation of sedimented organic matter [22]–[24]. The depth and extent of the hypoxic and anoxic layers depend on productivity and the frequency of deep-water renewals that occur during the fall and spring when dense, cold, oxygenated waters enter at depth over the sill at the mouth of the Inlet [25], [26], [27]. Depths below 100 m typically contain 1.0 ml/l of oxygen for most of the year [19]. This natural cycle of annual hypoxia and renewal is well documented in the sedimentary record in both recent past and throughout much of the Holocene [28], [29].

The renewal of oxygen in Saanich Inlet is ultimately dependent on oceanic conditions offshore of Vancouver Island, in the north Pacific?[10], [30]. In 2006, the continental shelf from Oregon to Washington suffered a severe hypoxic event causing massive mortality of demersal fish and benthic invertebrates as shallow as 50 m [5], [16]. Deep-water renewal in the neighboring Strait of Georgia (Figure 1) is derived from these same shelf waters that now experience repeated hypoxia [31]. Changes in the surface waters in the subarctic Pacific over the last 50 years [10], [32] have created a more buoyant surface layer that weakens gas exchange between the atmosphere and ocean interior [33]. As the northeast Pacific shows signs of increasing hypoxia, Saanich Inlet offers a natural laboratory to explore how benthic communities may respond to long-term changes and thereby understand the consequences of a shift in oxygen regimes at a broader scale.

In this study, we examined the dynamics of an epibenthic community in Saanich Inlet that occurs on both soft and hard substrata well into the suboxic zone [19], [34], [35]. We studied temporal changes in animal abundances on mud substratum and estimated temporal variations in area coverage by mats of sulphide oxidizing bacteria (*Beggiatoa* spp.) that occupy extensive areas of seafloor in Saanich Inlet below 100 m depth, fluctuating in coverage with the annual cycle of anoxia and deep-water renewal [36]. These bacteria grow at oxic-anoxic interfaces where they oxidize reduced sulphide with molecular oxygen. We used mat density as a proxy of biogeochemical processes as they reflect the presence of upwardly diffusing sulphide from subsurface anoxic sediments, and the availability of some dissolved oxygen in the benthic boundary layer. We expected bacterial mats to develop by the end of the summer after oxygen depletion and last until the first deep-water renewal event. We hypothesized that oxygen concentrations would drive changes in biodiversity following the respective physiological limitations of the different species, starting with the disappearance of benthic fish, and then crustaceans [12], [18].

The objectives of this study were to (i) determine species tolerances to hypoxic conditions, including the nature of response; and (ii) examine species replacement patterns across seasons and changing oxygen levels. The many published reviews summarizing the impacts of marine hypoxia e.g. [2], [11], [12], [37], [38] have usually looked to laboratory studies for information on thresholds of hypoxia for individual species [17], [18], [38] and to field sampling studies to evaluate community-scale effects [15], [16], [39]. Here we use high resolution *in situ* observations for direct determination of oxygen thresholds and behavioural responses of species and populations to varying oxygen levels.

## Results

### 1. Environmental Data

**Figure 2:**
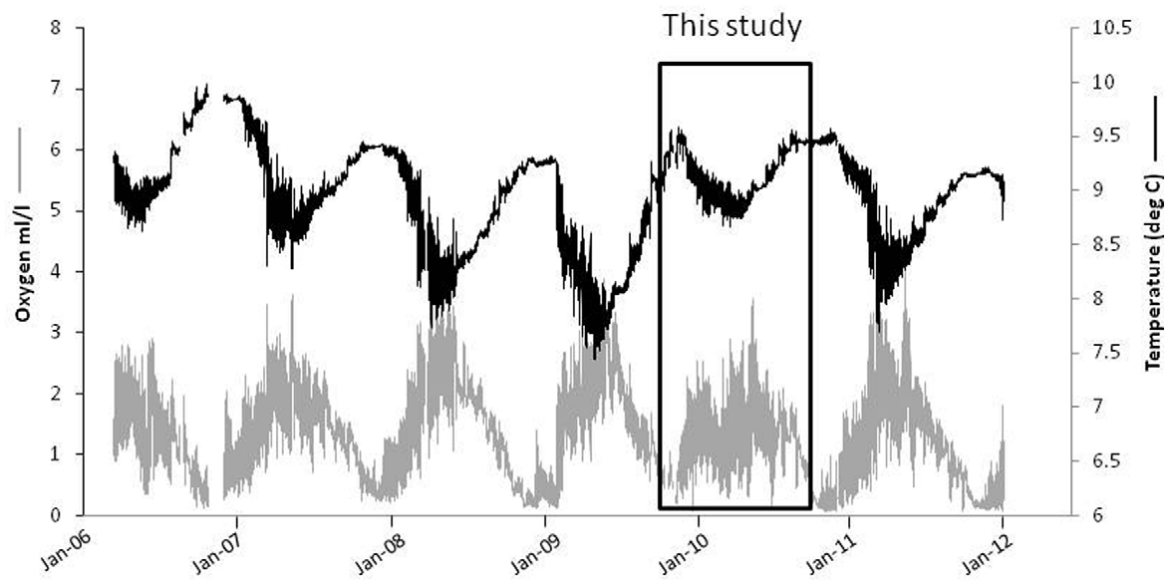
Temperature and dissolved oxygen recorded at the 95 m seafloor instrument platform (VIP) on VENUS in Saanich Inlet. Black line = temperature and grey line = oxygen. The period of this study is circumscribed by the box.

The six year record from the VENUS (Victoria Experimental Network Under the Sea) Saanich VIP station reveals that the study period was anomalous with respect to the typical annual pattern (Figure 2). Strong deep-water renewals usually push cold oxygenated water past the sensors starting in January with another injection in the boreal spring. The renewal began, as might be predicted, in early 2010 but was not sustained, resulting in spring/summer temperatures near 9°C and dissolved oxygen levels that rarely exceeded 2.4 ml/l (versus 3.0 ml/l in other years). In addition, heavy rains in the region induced notably fresher water such that bottom water densities in May 2010 were the lowest values of the entire record (1023.5 kg/m^3^).

**Figure 3:**
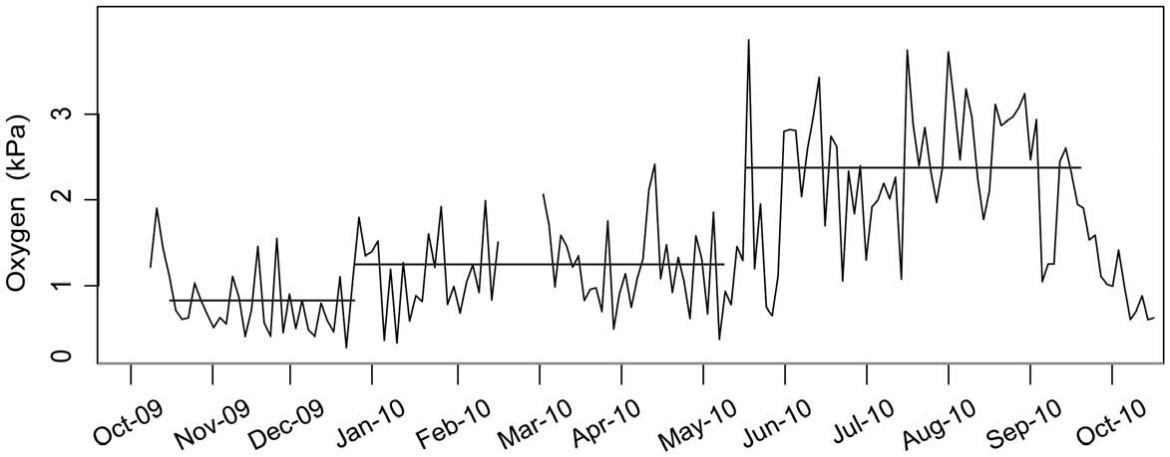
Dissolved oxygen levels at 102 m depth on the camera platform (DCF) in Saanich Inlet for the time of the study. Plotted values are averages of the measurements every minute in the hour encompassing the image capture. Horizontal bars are mean values representing three distinct periods of progressively less severe hypoxia, from October 13, 2009 to January 1, 2010 (average O_2_ = 0.81 kPa), from January 4, 2010 to May 3, 2010 (average O_2_ = 1.24 kPa) and from May 5, 2010 to September 20, 2010 (average O_2_ = 2.17 kPa).

Hypoxic conditions prevailed during the 09/10 study period, with hourly averaged dissolved oxygen levels varying between 0.31 and 3.96 kPa (0.09 to 1.32 ml O_2_/l) (Figure 3). Variability was high, with up to 1.1 ml/l changes within a single hour. Dissolved oxygen partial pressure was low at the beginning of the observation period ranging between 0.59 and 1.27 kPa (0.19 to 0.4 ml O_2_/l). Minor oxygen intrusions occurred in November leading to a generalized oxygen increase that remained in place from December through the spring. Beginning in May and throughout the summer, dissolved oxygen levels (and variability) increased again with a final decline into the fall of 2010 (Figure 3). Two oxygen intrusions distinguished three distinct periods of oxygen levels (ANOVA, F = 86; p<0.001) with the highest levels occurring in the summer (to 3.96 kPa) (Figure 3). Temperature and salinity (salinity not shown) showed low variability over the entire period with values ranging from 8.9 to 9.5°C and 30.4 to 31.2 psu, respectively.

### 2. Bacterial Mat Coverage

**Figure 4:**
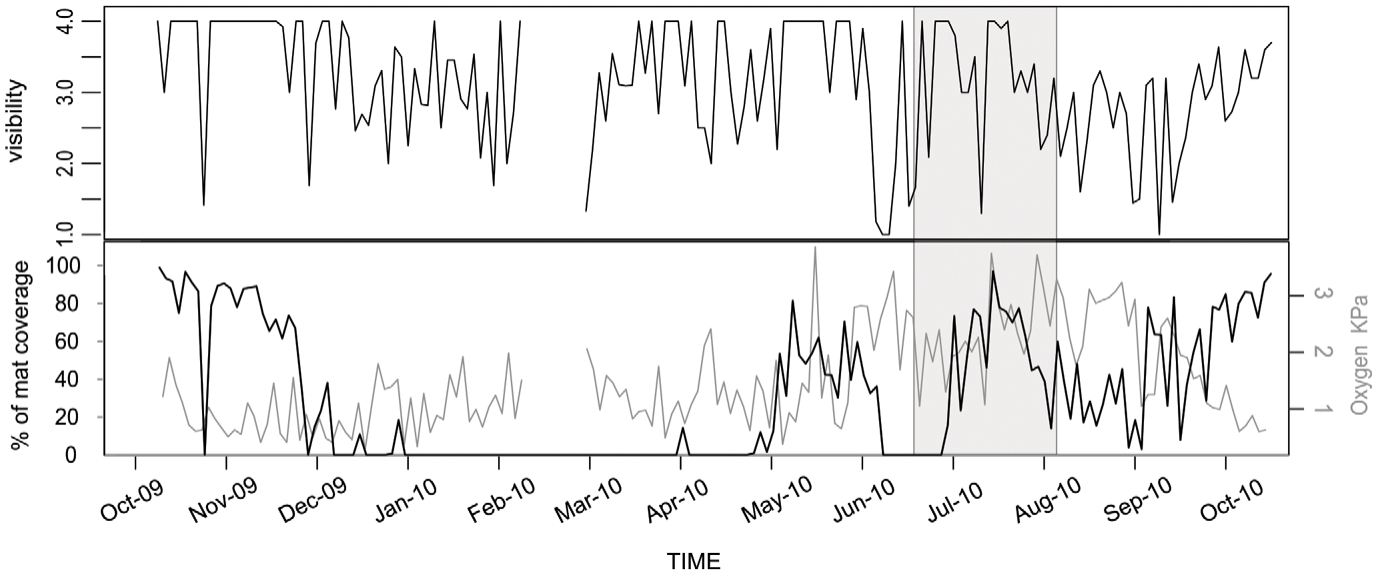
Time series of relative abundance of bacterial mats and visibility between October 6^th^ 2009 and October 18^th^ 2010 in the camera field of view in Saanich Inlet. Top panel is visibility and lower panel bacterial mat coverage. The grey box shows the period characterized by the presence of phytodetritus on the seafloor. The grey line is the partial pressure in dissolved oxygen.

**Figure 5:**
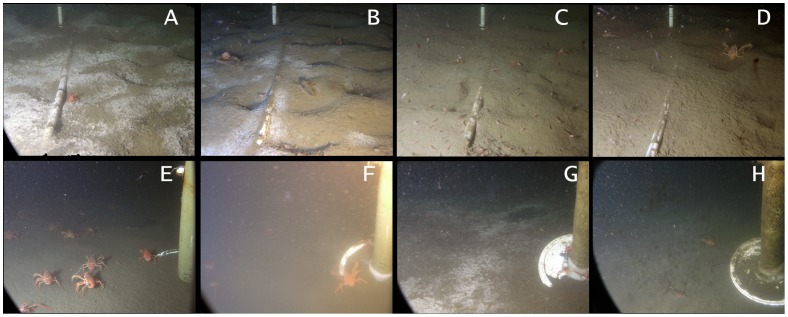
Photos of study area at the Digital Camera Platform VENUS location in Saanich Inlet. The pictures show bacterial mats and megafauna. A. Oct 6′09– Medium *Munida* on dense mat; thick bands on white scale are 10 cm apart. B. Nov 18′09– Tiny *Spirontocaris*, large *Munida*, *Lyopsetta* in pits created by the flatfish (and a fir cone); note diminishing mat. C. Dec 14′09– Maximum *Spirontocaris* abundance; medium *Munida* and one *Lyopsetta* in the image. D. Feb 12′10– Large *Munida* on a smoothed seafloor with no mat; zooplankton include euphausiids and amphipods. E. Apr 1′10– Abundant large and medium *Munida* beside camera leg. F. May 28′10– Image illustrates a turbid event (caused by resuspended sediment) that obscures distal areas. G. Jul 10′10– Seafloor has diatom layer and bacterial mat; the black area is lifted mat that reveals likely sulphidic sediment. Small Munida on camera foot where animal activity has cleared the sediment. H. Sep 24′10– Visibility now reduced by biofilm on camera.

Bacterial mats extensively covered the seafloor at the beginning of the observation period in October and November 2009 when dissolved oxygen concentrations were low (Figures 4 and 5), then abruptly disappeared at a rate of 2.54% coverage per day between November 13^th^ and December 18^th^ 2009. Sparse mats reappeared during late spring-early summer (i.e. 8.1% coverage per day between April 18^th^ and April 28^th^ 2010) and, to a greater extent, during the summer (i.e. 4.6% coverage per day between June 18^th^ and July 10^th^ 2010) when, in contrast with the October-November 2009 situation, sensors measured the highest oxygen concentrations of the year (i.e. between 2 and 3.9 kPa). During the latter period accumulations of phytodetritus was visible on the seafloor and on the camera tripod.

### 3. Species Patterns

**Figure 6:**
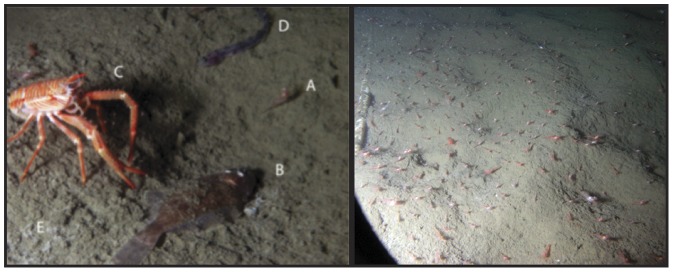
The common species recorded by the camera on VENUS in Saanich Inlet. The right image (Nov. 25, 2009) with *Spirontocaris* (A), *Lyopsetta exilis* (B), *Munida quadrispina* (C), *Plectobranchus evides* (D) and traces of bacterial mat (E). The left image with numerous *Spirontocaris sica* was taken on Dec 09, 2009. Images about 20 and 30 cm across.

The epibenthic community in the study area in Saanich Inlet was dominated by three species: the galatheid squat lobster *Munida quadrispina*, the pleuronectid flatfish *Lyopsetta exilis* and the hippolytid shrimp *Spirontocaris sica* (Figure 6). These species represented 98% of all individuals counted. Other species occasionally present in the field of view included: bluebarred prickleback *Plectobranchus evides*, Pacific herring *Clupea pallasii*, zoarcid eelpout *Lycodopsis* sp., Pacific hake *Merluccius productus*, walleye pollock *Theragra chalcogramma,* blacktip poacher *Xeneretmus latifrons* and, rarely, the commercial spot prawn *Pandalus platyceros* and scorpaenid rockfishes. A total of 12 taxa and 17,988 observed individuals (13,570 of *S. sica* alone) were recorded over the study period in 2,178 images.

**Figure 7:**
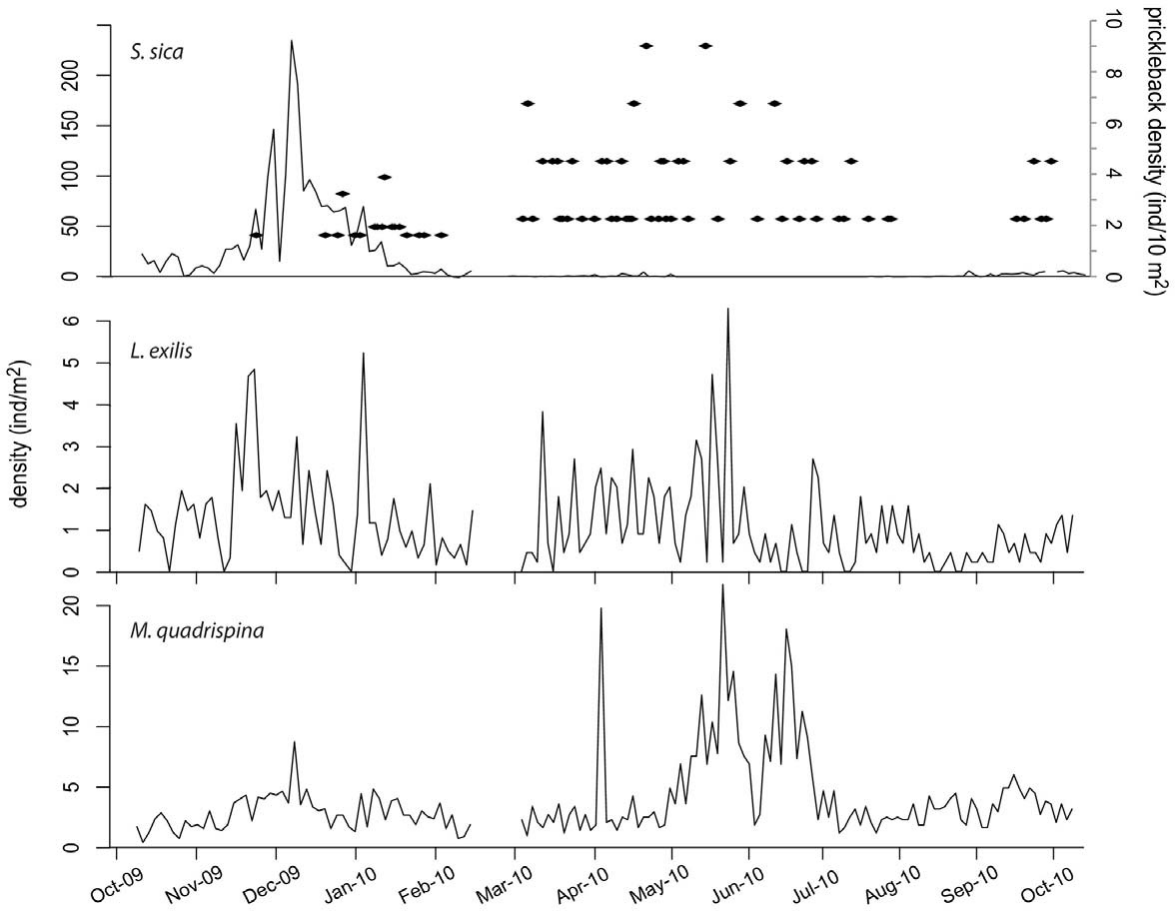
Time series of species densities between October 2009 and October 2010 in the camera field of view in Saanich Inlet. The shrimp *Spirontocaris sica* (black line) and the prickleback *Plectobranchus evides* (black dots) (upper); the flatfish *Lyopsetta exilis* (middle); and the squat lobster *Munida quadrispina* (lower). Note the variation in density scale among species.

**Figure 8:**
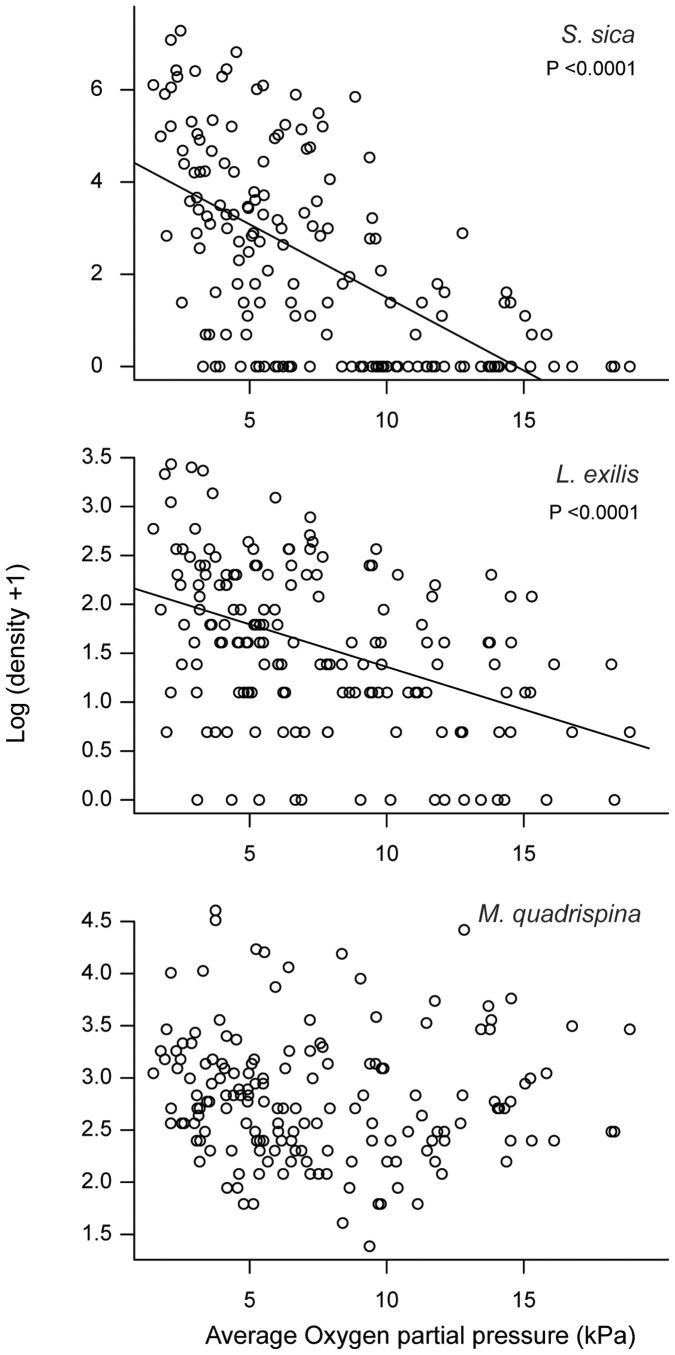
Species abundances relationship with DO concentration’s at the camera site in Saanich Inlet. Relationship between hourly mean oxygen concentration and species abundances for the shrimp *Spirontocaris sica* (upper), the flatfish *Lyopsetta exilis* (middle) and the squat lobster *Munida quadrispina* (lower) in Saanich Inlet.

*Spirontocaris sica* was the most abundant species between November 2009 and January 2010 (Figure 7). In videos, individuals were swimming above the bottom, alighting on the seafloor, and stationary in large groups. At the beginning of December 2009, densities exceeded 200/m^2^ (Figure 6). Image and video observations suggest that this species aggregates on bacterial mat patches rather than on bare sediment areas. A sharp decrease in shrimp density of 4 individuals per m^2^ per day was coincident with the complete disappearance of bacterial mats at the end of December. After late April, they were absent until late August when they began increasing in density. *S. sica* was abundant at dissolved oxygen concentrations as low as 0.3 kPa (0.1 ml/l), revealing a high tolerance to severe hypoxia (i.e. <0.5 ml/l, [2]). There was a significant negative relationship between *S. sica* density and the average dissolved oxygen partial pressure (Adjusted-R^2^ = 0.35; p<0.0001; Figure 8).

Coincident with the disappearance of the bacterial mats in mid-December was the first appearance of the bluebarred prickleback *Plectobranchus evides* which remained in small numbers (total 109 seen) until the end of the study period. Sightings of multiple prickleback were most common between April and July.

A total of 924 individuals of the flatfish *Lyopsetta exilis* were counted during the study period. *L exilis* was usually resting on the bottom, sometimes half or entirely buried in the sediment. With abrupt movements, these fish caused sediment resuspension events. Abundance peaked at intervals over many months from October 2009 to June 2010 when abundance diminished (Figure 7). There was a significant negative relationship between *L. exilis* densities and oxygen partial pressure (Adjusted-R^2^ = 0.18, p<0.0001; Figure 8).

The squat lobster *Munida quadrispina* was the second most abundant species observed in the area. A total of 3,106 individuals were observed walking through the field of view, resting or deposit feeding on the sediment surface or occasionally catching zooplankton in the water column. *M. quadrispina* abundance was constant through much the year with peaks in density during late spring (Figure 7). The dip in density visible in early June in the data was likely due to poor visibility affecting the counts. There was no significant relationship between density and dissolved oxygen partial pressure (Figure 8).

**Figure 9:**
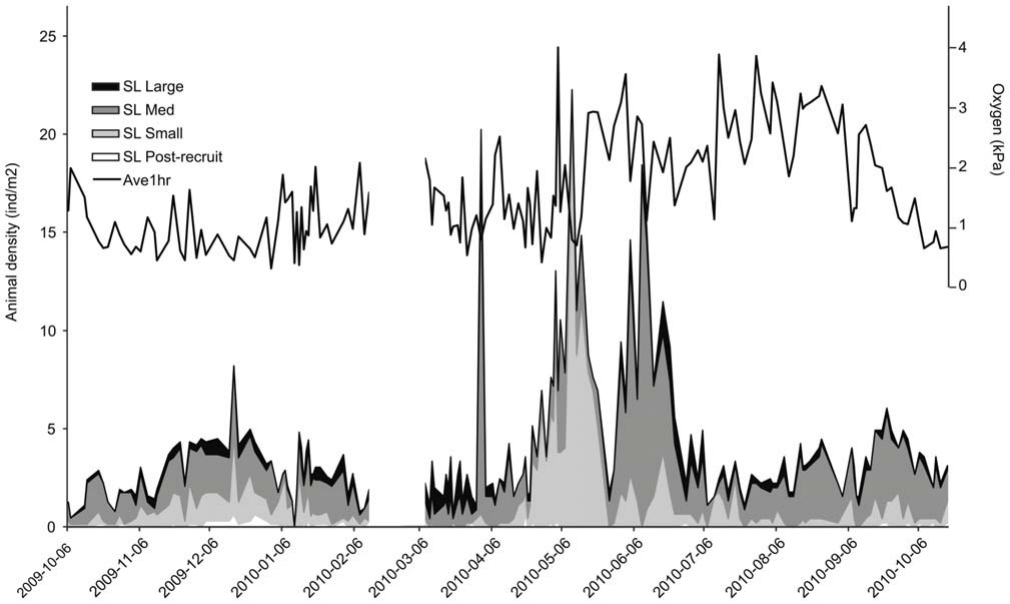
Densities of *M. quadrispina* life stages between October 2009 and October 2010 in the camera field of view in Saanich Inlet. The black curve depicts the levels of oxygen saturation and the black lines the presence of bacterial mats. The notable abundance decrease around June 1^st^ occurs at a time of very low visibility and may be an artifact.

*Munida quadrispina* size distribution varied over the observation period. Some tiny post recruitment individuals appeared from December 2009 to March 2010 (mostly visible only in the near field). Large individuals were constant in numbers throughout the year while peaks in small and medium-sized squat lobsters were observed in April-May 2010 and June-July 2010, respectively. Overall, small squat lobster abundance tended to be lower when dense mats (i.e. >90% coverage) were present (Figure 9). The marked decrease in medium sized squat lobsters coincided with the appearance of phytodetritus on the bottom (Figure 4).

### 4. Community Characteristics

In order to understand factors controlling the epibenthic community, we considered the relative influence of the absolute oxygen concentration and its range of variation in the Redundancy Canonical Analysis and Principal Coordinates of Neighbor Matrices, used to describe the temporal structure of the three dominant species (Table 1). Altogether, the nine environmental variables quantified in this study explained 67.9% of the variation over the entire period. The three sub models identified from the significant PCNM functions (see section 5 in methodology) were: (i) one month-broad scale (8 significant PCNM for the group 1 to 8; R^2^ = 0.328), (ii) two weeks-medium scale (11 significant PCNM for the group 9 to 21; R^2^ = 0.410), and (iii) one week-fine scale (7 significant PCNM for the group 23 to 47; R^2^ = 0.018). Dissolved oxygen concentrations, represented by several variables, significantly contributed to each sub-model (Table 1). The standard deviation, minimum value and range were the most important at the broad scale, while the maximum value and range emerged at fine scale. These results suggest that variability in oxygen concentrations was more important than the absolute value for the three species present. At medium scales, the main drivers were temperature and visibility.

**Table 1:** Results of the Principal Component of Neighbor Matrices (PCNM).

	All	Broad	Medium	Fine
R^2^ submodel on 3 species	–	0.328	0.41	0.018
R^2^ environment on submodel	–	0.382	0.582	0.078
R^2^ environment on 3 species	0.679	0.125	0.239	0.001
O_2_ average (kPa)	0.550	0.1997	0.3797	0.3294
O_2_ Standard deviation (kPa)	0.010*	0.0027**	0.5625	0.0686
O_2_ minimum (% Sat)	0.610	0.0372**	0.3634	0.1045
O_2_ maximum (% Sat)	0.105	0.4393	0.8025	0.0009**
O_2_ range (% Sat)	0.005**	<0.0001**	0.0825	0.0002**
O_2_ median (% Sat)	0.270	0.3966	0.3476	0.3707
Salinity (psu)	0.480	0.5022	0.2942	0.1632
Temperature (°C)	0.005*	0.7131	0.0004**	0.5121
Visibility	0.340	<0.0001**	0.025*	0.3856

The PCNM was conducted on the 3 main species encountered in the study area (Squat lobster *Munida quadrispina*, the flatfish *Lyopsetta exilis* and the shrimp *Spirontocaris sica*). Values in the lower part of the table are the exact p-values (* p<0.05, ** p<0.01).

**Figure 10:**
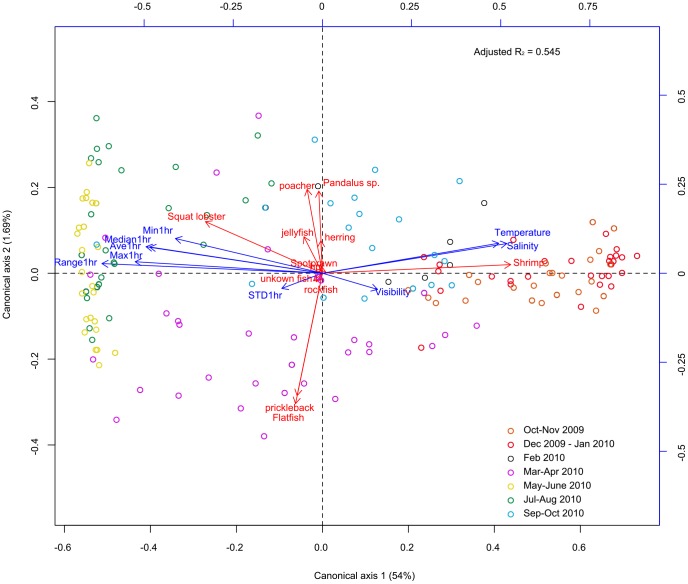
Redundancy analysis correlation biplot of the observations dates, species and environmental variables. Saanich Inlet. Canonical redundancy analysis is based on the transformed species abundance of macrobenthic communities reported in the camera field of view in Saanich Inlet. Ave 1 hr: hourly average, Max 1 hr: hourly maximum, Median 1 hr: hourly median, Min 1 hr: hourly minimum, Range 1 hr: hourly range, STD1hr: hourly standard deviation.

At the broad community level, the results of the forward selection RDA (Figure 10) showed that transformed species abundances were significantly correlated with four environmental variables: range, standard deviation and maximum value of dissolved oxygen concentration, and the temperature (adjusted R^2^ = 0.54, p = 0.001). The first and second axes accounted for 54% (p = 0.001) and 1.69% (p = 0.007), respectively, of the total variation in species abundance data. The first canonical axis separated two main groups: one included observations dates between October 2009 and February 2010 (Fall 2009–Winter 2010) and the second, observations dates from May 2010 to August 2010 (Summer 2010) on the right and left side of the axis respectively. Observations made in March-April 2010 and September-October 2010 were intermediate between those two groups. Observations in summer 2010 were associated with higher maximum values and higher variability, in dissolved oxygen concentrations measurements, and lower temperature.

## Discussion

### 1. Methodology

On-line image acquisition delivered a long term, high-resolution time series with daily information on changes in community dynamics. This approach has limitations. The camera tripod could have attracted bentho-pelagic fish and benthic fauna, although a study of such structural effects in the deep North Pacific found no influences on megafaunal abundances [40]. Even if such effects were present, the light and infrastructure were constant throughout the study. We are thus confident that changes observed in species density are linked to internal or external factors other than the presence of the observing system. Light may, however, have introduced artefacts in the bacterial mat studies. Fish were sometimes attracted by the light, and their swimming activity disturbed the sediment surface colonized by the bacterial mats possibly resulting in an underestimation of coverage estimations. The value of direct field observation outweighs these shortcomings, as noted by a recent study of bioturbation responses to hypoxia [41].

We also considered the possibility of an uneven temporal distribution of error in our counts of organisms. Constant camera settings, lighting and pan/tilt coordinates assured comparability of images acquired by several operators. The main factor that affected accuracy in counts was visibility. During periods of low visibility caused by resuspended sediments [42] (Figure 5F), animals located in the back of the field of view were not visible. This might partly explain the significant statistical influence of visibility on animal counting, especially following the spring bloom.

We applied multivariate ordination methods to determine temporal structures in our dataset. The principal coordinate of neighbour matrices was developed to identify spatial ecological patterns and their relative importance in terms of adjusted variance explained [43]. This method has seen little use in ecological time series but has significant advantages over time lag analysis [44]. First, the irregularity in the time-series made the use of traditional time series analysis difficult. But more importantly, the RDA-PCNM revealed different patterns of temporal changes and the contribution of each species and environmental variables to those patterns; it also provided information on the amount of variance explained by the identified model [44].

### 2. Environmental Conditions

Throughout the study period, hourly averaged dissolved oxygen concentrations were typical of hypoxic conditions (i.e. 1.4 ml.l^−1^ ≈ 4.5 kPa; [1]). The observed oxygen intrusions, although weak, were characteristic of renewal events that occur annually in Saanich Inlet. Dense oxygenated water enters over the sill replacing deep anoxic water that oxygenates upward; past studies describe a single boreal fall phenomenon (e.g. [24], [25]). The source of renewal is a complex interaction of deep dense water from the continental shelf mixing through sills in the outer Strait of Georgia and the outflow of the Fraser River that results in two main pulses of deep-water renewal into the Strait of Georgia and adjacent inlets [45]. Tidal forcing also pumps water masses inland during neap tides causing peaks in the temperature (and oxygen) signal [45]. Manning et al. [26] are the first to explicitly identify the arrival of two renewal events into Saanich during spring and fall of 2008 and link water mass behaviour to Strait and shelf phenomena. VENUS data over several years confirm the presence of additional spring density events (data not shown).

During our study, however, the Saanich “renewal event” was significantly weaker compared to previous years (Figure 2). Unusually cool water dominated the BC coast in 2007 through 2009 as reflected in the general cooling trend in Saanich Inlet culminating in a La Niña winter in 08/09 followed by a relatively strong El Niño event initiating in 2009 [46]. By March 2010, the water offshore Canada and USA was warmer than normal and sustained southerly winds inhibited upwelling [47]. Thus, in the spring of 2010, there was little cool deep-water influx through the Straits to Saanich and water remained warm with little additional oxygen through the spring months. Fauna at 100 m would normally experience dissolved oxygen concentrations of over 2.5 ml/l but even 1.5 ml/l was rare this year (Figure 2).

### 3. Bacterial Mats

Anoxia and H_2_S concentration act together on benthic communities and, while their individual effects are difficult to separate [36], it is important to consider H_2_S when studying the response of benthic organisms to hypoxia/anoxia [48]. No H_2_S sensor was available for this study and the VENUS oxygen sensor was deployed 1.5 m above the bottom. We used the presence of the mat-forming, sulphide-oxidizing bacterium *Beggiatoa* sp. as a proxy indicator of redox conditions at the seafloor. Because these bacteria require both substances for their energy metabolism, mats usually form an interface between hydrogen sulphide diffusing upward from the sediments and dissolved oxygen in the overlying bottom water [49]. Beyond the identification of this redox boundary, the presence of *Beggiatoa* sp. mats on the sediment surface is also indicative of hypoxic conditions in bottom waters favorable to their micro-aerophilic metabolism [49]. Micro-electrode profiles in cores from the camera site in previous years have shown that even when the bacterial mats are absent, the redox boundary occurs at 1–2 mm below the sediment surface (Juniper, unpub. data).

Our observations suggest that bacteria mat grows over a wide range of hypoxic bottom water oxygen concentrations. In the fall, mats were abundant at the lowest measured oxygen levels. Yet in the spring, and especially in the summer, *Beggiatoa* sp. mats developed while the oxygen sensor was detecting the highest oxygen concentrations of the year (4 kPa). An increased supply of hydrogen sulphide from decomposition of fresh organic material could compensate for higher dissolved oxygen levels in overlying seawater that could be detrimental to *Beggiatoa* spp metabolism. Redox boundaries within these bacterial mats are determined by a dynamic balance of oxygen diffusing from above and hydrogen sulphide from below. This explanation fits our observations of the simultaneous presence of phytodetritus, bacterial mats and milder hypoxia during the spring and early summer phytoplankton bloom season in Saanich. 16S rRNA gene data from a single mat sample collected by submersible confirmed that the dominant organism belonged to the genus *Beggiatoa*. The sequence data from this sample grouped closely with that of *Beggiatoa* sp. from the Bay of Concepcion and *Thioploca chileae* (SK Juniper, unpublished data).

### 4. Species Patterns

Three species were very tolerant of sustained low dissolved oxygen concentration. Similar tolerances occurs in the epilithic community of Saanich Inlet at the anoxic interface [19]. Hypoxia thresholds vary among sediment benthic organisms with polychaetes and mollusks the most tolerant, followed by crustaceans, echinoderms and fish [12], [18]. In this study, although small organisms were difficult to observe with the camera, the three dominant species included two decapod crustaceans and a flatfish. Polychaetes and a lucinoid clam were present but rare in ROV push-cores from the vicinity (Tunnicliffe, pers. obs.).

The shrimp *Spirontocaris sica* was only present during the periods of lowest oxygen concentration between October 2009 and January 2010. It commonly appears in zooplankton hauls along the coast from California to British Columbia at depths ranging from 87 m to 848 m [50]. In this study, this species was observed ‘resting’ on the seafloor suggesting a bentho-pelagic life mode. This first record of a benthic mode is supported by video observations in which *S. sica* landed on the seafloor slowly swimming down from the water column, often in large numbers. High resolution ROV video imagery also records this species feeding on *Beggiatoa* mats. These shrimp may also benefit from sulfide detoxification by bacterial mats as previously suggested for the squat lobster *Pleuroncodes monodon* off Chile [51]. Its abrupt departure from the camera site in January coincided with the disappearance of the bacterial mats and the arrival of potential predatory fish species in the area, including the blacktip poacher *Xeneretmus latifrons*, the Pacific hake *Merluccius productus* and the walleye Pollock *Theragra chalcogramma*. ROV video observations in February 2010 recorded *S. sica* in deeper water between 112 and 117 m depth where oxygen concentrations were ranging from 0.2 to 0.3 ml/l (Matabos, pers. obs.). However, the relative influences of food availability, avoidance of predation or bottom disturbance on shrimp populations when greater oxygen concentration brings more fish, remain to be determined.

The flatfish *Lyopsetta exilis* showed significantly higher densities during periods of severe hypoxia. This species is commonly reported from bottom trawl surveys along the west coast of North America from California to Vancouver Island [52]–[55] in areas regularly fed by low oxygen waters upwelled from the Pacific oxygen minimum zone. While most northeast Pacific demersal fishes species decrease in abundance with decreasing oxygen, the distribution of *L. exilis* appears independent of oxygen concentration along the coast [16]. *L. exilis* is planktivorous with a diet mostly constituted of pelagic crustaceans including euphausids, and pelagic shrimp; off the Oregon coast, they mainly feed on *Pandalus jordani*, but also occasionally on shrimp from the genus *Spirontocaris* [53]. While no *Spirontocaris* were found in the few *L. exilis* individuals examined from Saanich Inlet, stomach contents analysis confirmed a pelagic feeding habit (Tunnicliffe, unpub. data). In Saanich Inlet, the deep scattering layer follows the depth of the hypoxic contours [56] which may play an important role in the behaviour and distribution of *L. exilis*. *L.exilis* migrate in Saanich as the anoxic boundary shifts [42]. This migration likely occurs in response to changing zooplankton concentration at the intersection of the seafloor and the hypoxic zone over the year. This species is important in bioturbation of a seafloor with low infauna, resuspending sediment and facilitating nutrient recycling [42]; we see a marked change in seafloor roughness throughout our study. It is very likely the fish activity aerates sediments and diminishes the growth potential of bacterial mat.

In Saanich Inlet, the galatheid crab *Munida quadrispina* is very tolerant of hypoxia, with high densities found at oxygen levels as low as 0.1 to 0.15 ml/l (this study; [34]). However, no significant relationship was found between the oxygen partial pressure and the squat lobster density suggesting that at these low levels of oxygen, *M. quadrispina*’s abundance is not affected by oxygen. The gill mass of individuals living in hypoxic conditions exceeds that of those in oxic conditions [57] suggesting a local adaptation. We saw mostly low activity and no aggressive behaviour during this study as noted by Burd & Brinkhurst [34]. Childress & Siebel [20] noted reduced activity for a number of benthic marine species in hypoxia.

A study the preceding year at this site noted settling recruits through Nov-Dec of 2008 [58] as found in our study. Throughout our observations, all sizes of *Munida* were present; while we could not collect exact size data, the strong relationship of size with oxygen found on Saanich rock walls [34] was not evident on the mud. Small squat lobsters appeared in abundance at the onset of increased oxygen in mid-May 2010. In this study, however, the disappearance of small squat lobsters was not related to oxygen concentration: they disappeared while the hourly average concentration was increasing. Juveniles of most marine invertebrates species have lower tolerance limits than adults [59] thus the abundance peaks in smaller squat lobsters may relate to high oxygen incursion in May and June. However, the subsequent decrease in abundance in July is not easy to explain. Late June/July was the only period in which rockfish and several other fish species appeared; the squat lobsters may have migrated away from these predators. Here, small squat lobsters may be less tolerant of high frequency variations in dissolved oxygen. Release of H_2_S from the sediment could also be an important factor. Indeed, survival times of benthic organisms under hypoxia are reduced by sulfide exposure [60].

Under induced hypoxia, crustacean species change their behaviour (e.g. abandon shelter, alter activity patterns) leading to changes in intra- and inter- specific interactions; when hypoxia reaches a critical level, species become immobile and eventually die [38]. We did not observe moribund behaviour, probably because sustained natural conditions in Saanich Inlet have fostered an adapted community. However, we noted few interactions among organisms in video sweeps. *Munida quadrispina* exhibits territorial and aggressive behaviour under normoxic conditions but remained passive at the oxygen levels in this study.

All common species were highly tolerant to hypoxia and severe hypoxia. The flatfish *Lyopsetta exilis* and the shrimp *Spirontocaris sica* even showed a significant negative relationship with hourly average oxygen partial pressure with high abundances near anoxia. When oxygen concentrations increase, these species probably migrate deeper where competition and predation are low and food supply remains high as zooplankton also migrate deeper. This behaviour is known in oxygen minimum zones where tolerant species take advantage of abundant food supply [21]. Conversely, periods when the highest concentrations of oxygen were recorded also coincided with higher daily and even hourly variability in oxygen concentration (data not shown). The absence of relationships between the density of the squat lobster *Munida quadrispina* and oxygen partial pressure in the range recorded suggests that this species is able to cope with high frequency variability in oxygen concentrations, as revealed by the RDA where observations made between May and August were associated with large ranges in oxygen concentration and a dominance of squat lobsters. This variability may impose physiological limitations on flatfish and shrimp. The data do not allow assessment of the role of variability in oxygen concentration on species distribution and behaviour. Laboratory studies and higher resolution *in situ* observations are required. Vertical migration during unobserved periods to repay oxygen debt is unlikely for squat lobsters; we do not record diel migrations. Occasionally, flatfish are observed several meters off bottom [42] possibly feeding or reducing sulphide contact. A previous study conducted with the same camera in the same year showed that there is no daily variation in the abundance of the three dominant species [61].

While neither CO_2_ concentrations nor pH measurements were available for this period, higher CO_2_ concentrations and consequent increases in acidity usually accompany low oxygen conditions [62]. Increased pCO_2_ and/or decreasing pH modify organism extra-cellular acid-base balances and can reduce oxygen affinity, thus amplifying physiological constraints on organisms [63].

### 5. Community Responses to Seasonal Changes: Implications at Larger Scale

Multivariate analysis detected patterns in community composition at different temporal scales from a week to a month. Variability in dissolved oxygen partial pressure (measured as the range and standard deviation) and temperature explained 38% of the one-month broad scale pattern, and is likely related to changes in tidal regimes [26]. The most important temporal component detected in community structure was the fortnightly pattern, which was highly correlated with temperature and visibility. Given the very low variations in temperature values over the year, the two-week structure in the data might reveal other processes occurring over a tidal fortnightly cycle. Currents at the study site are very low with low variability (averaging less than 5 cm/s; [42]), but likely contribute to mixing processes. Visibility changes due to sediment resuspension mostly because of flatfish activity [42], [64]. During spring tides, increasing tidal mixing outside the fjord causes seaward, flow of surface waters and nutrient-rich sub-surface waters to flow inward [24]. This nutrient injection appears to support fortnightly productivity peaks [23], [24]. We propose that the benthic community responds to this tidal forcing through changes in physico-chemical parameters and in the abundance and activity of zooplankton that feed on the enhanced phytoplankton. This latter link requires further investigation. Finally, at fine scale the macrofaunal benthic community may be structured by biotic processes such as prey/predation interactions, or competition.

**Figure 11:**
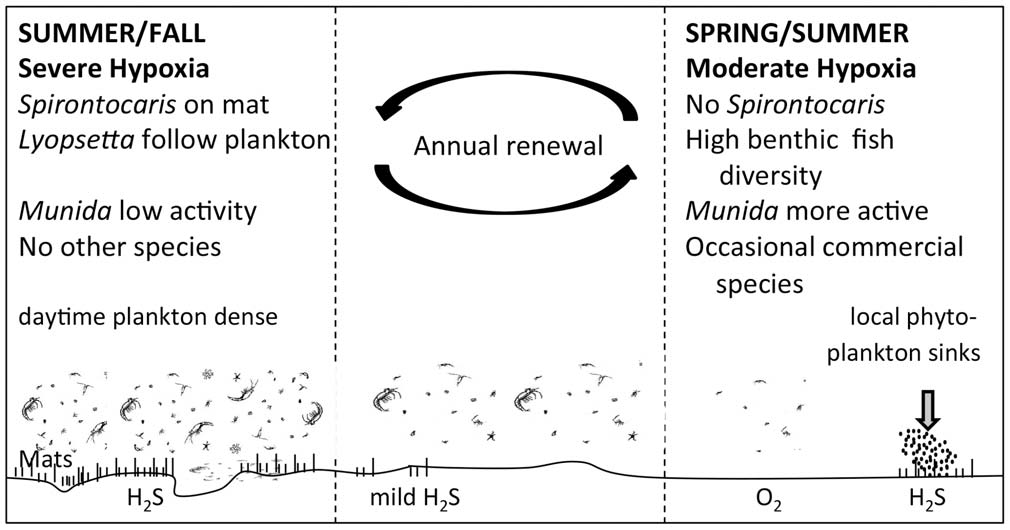
Observations and underlying processes occurring at different levels of hypoxia in Saanich Inlet. Diagram summarizes seasonal changes and effects on benthic communities inhabiting the hypoxic zone of Saanich Inlet (BC, Canada). Gradual depletion of oxygen in the basin following the spring phytoplankton bloom leads to the development of bacterial mats on the seafloor. Changes in oxygen indirectly affect the benthic community through: i) food availability by controlling access to downwardly migrating zooplankton, and the growth of bacterial mats on the seafloor, and ii) disturbance/predation with greater activity occurring in higher oxygen concentrations. Note that H_2_S can be released during moderate hypoxia in response to local bacterial decomposition in surface sediments (e.g. following a plankton bloom).

The RDA results revealed seasonality in community composition, mainly driven by dissolved oxygen concentration changes. Variations in oxygen at the different temporal scales in Saanich Inlet can be complex and substantial, and strongly related to the different tidal components controlling water masses movements, and the annual deep-water renewals occurring in the fall and spring [26]. Even if the underlying mechanisms are complex, these variations in oxygen have a strong influence on benthic community dynamics, directly through species physiological tolerances or indirectly by controlling food availability (zooplankton migration and bacterial mats). Figure 11 summarizes the observations made in varying hypoxic levels and the processes shaping benthic community structure in Saanich Inlet.

Higher species richness occurred during periods of higher oxygen concentration in spring and summer. Several fish species (e.g. dogfish, rockfish) and commercial crustacean species like the spot prawn, *Pandalus platyceros* and the Dungeness crab, *Metacarcinus magister* normally inhabit these depths but do not tolerate severe hypoxia. Their occurrence during milder hypoxia underscores the value of high-resolution *in situ* observations for determining hypoxia thresholds of species in their ecological context. Loss of biodiversity can have important effects on ecosystem function and energy pathways [13], [65]. For example, during the first period when dissolved oxygen concentration level was ∼0.8 kPa (0.25 ml/l, Figure 3), the three main tolerant species fully replaced all other benthic species (only four individuals of a different species were seen). The consequences of such species exclusions by hypoxia depend on the degree of species functional redundancy or complementarity. If the tolerant species has similar influences on energy fluxes (e.g. trophic level, biogeochemical cycle), ecosystem function can be maintained [66]. During periods of hypoxia, oxygen is quickly lost in the sediment and H_2_S released such that the redox potential discontinuity layer rises to the sediment-water interface. Under these conditions, infauna are eliminated and bioturbation strongly reduced [2], [67]. While this could be critical for geochemical cycling, this study shows that bioturbation is not totally compromised. In Saanich Inlet, the flatfish *Lyopsetta exilis* can resuspend 1.3±0.7 l bulk sediment/m^2^/d and thus plays a major role in organic matter remineralisation and nutrient recycling [42]. *Lyopsetta*, however, does not burrow deeply, thus functional replacement is not complete.

Our three dominant species, or close relatives, also occur in the Strait of Georgia and on the continental margin off Vancouver Island [31], [54] where long-term shifts in oceanographic conditions, including decreasing dissolved oxygen concentrations are observed [4], [10], [30], [31]. *Lyopsetta exilis* is found in high abundance on the continental margin in the North Pacific [52], [54]. As oxygen minimum zones grow and upwelling events intensify [6], [7], this flatfish could colonize large areas of the East Pacific coast. Similarly, range expansion may occur in the hippolytid shrimp *Spirontocaris sica* and *Munida quadrispina,* both of which are recorded at least from California to Vancouver Island [50], [68]. One consequence of an expanding oxygen minimum zone is the compression of habitat into shallow water as observed for pelagic fish [69] and groundfish [70]. Another impact may be increased predation as species no longer can retreat to deep, dark water [71]. Benthic habitats subject to chronic oxygen depletion can be particularly sensitive to local organic enrichment, as illustrated by the presence of bacterial mats at our study site during a period when near-bottom oxygen levels had actually increased as a result of inlet-scale water mass renewal (Figure 11).

The seasonal hypoxia-anoxia cycle in Saanich Inlet, plus the presence of a hypoxia-tolerant benthic community and the VENUS observatory provide an opportunity to understand how benthic communities may respond to such ocean change. While changes in dissolved oxygen concentration can directly affect benthic organisms depending on their physiological tolerance, their influence on community dynamics can be complex and indirectly influence benthic communities through a cascade of mechanisms. While restoration of coastal areas affected by anthropogenic eutrophication is possible at small temporal scales, an increase in hypoxic areas resulting from climate change is a non-reversible phenomenon given the momentum in ocean change currently underway. As hypoxia increases on the continental shelf, we predict a shift to low diversity, hypoxia-tolerant communities of lower commercial significance. By modifying community composition, life history stage distribution and interactions among species, these changes will have important implications for the effects of trawling and the delivery of ecosystem services. The study of naturally hypoxic systems will enhance our capacity to forecast consequences of growing hypoxia in coastal environments and upwelling areas, and thus inform better resource management.

## Methods

VENUS (Victoria Experimental Network Under the Sea) is a cabled seafloor observatory with arrays in both Saanich Inlet and the southern Strait of Georgia in British Columbia. The Saanich Inlet array includes a shore station at the Institute of Ocean Sciences (IOS) and a 3 km cable to a Node at 100 m depth in Patricia Bay (48° 39.05′N 123° 29.20′W, Figure 1). Data from numerous instruments are archived and available in near real-time while some instruments – such as the camera in this study – are operated directly by investigators; images are also archived. See www.venus.uvic.ca. Instruments are maintained twice a year; during this study, the camera was lifted, cleaned and redeployed occupying the same position within half a meter.

### 1. Environmental Characterization

Dissolved oxygen concentration and temperature were measured by instruments on a benthic platform (Digital Camera Frame, DCF) at 102 m depth (Figure 1). As part of the salinity record was absent at the DCF, we used salinity data from another platform (Venus Instrument Platform, VIP) at 94 m depth, about 135 m distant from the DCF. All measurements were made at 1.5 m above the bottom. Dissolved oxygen was measured once per minute in ml/l for the first time period using an Aanderaa Optode 0418 sensor and once per second in the second time period using a SeaBird 43 Oxygen 1238 sensor; intercalibration of the sensors was performed in the lab after the second deployment. Averaged values were calculated from +/− one hour bracketing the time of the image to smooth short-term variability. Other measures of dissolved oxygen were calculated to characterize the variability in dissolved oxygen concentrations: range, standard deviation, minimum and maximum values. For close examination of the study year, the Matlab functions *O2sol* and *vpress* (MathWorks Inc., 2011) were used to convert dissolved oxygen concentration in partial pressure (kPa), selected for its influence on biological processes and physiological performance [72], [73]. We have not considered the role of currents at this site as analyses of VENUS data show them to be negligible [42].

### 2. Image Acquisition: VENUS Camera

Images were collected using a remotely operated underwater camera (a modified Olympus C8080 wide zoom camera with an 8 megapixel CCD and an f2.4, 5x optical zoom lens: “Cyclops” by C-Map Systems Inc). A pan and tilt unit allowed complete seafloor coverage (+/−90° tilt and +/−180° pan) and illumination was available on demand from an Ikelite 200 Ws flash and three Deep Sea Power and Light 100 W incandescent lamps for still images and videos, respectively. For scale, the camera was equipped with two 10 mW red lasers with 10 cm separation. The camera system was mounted on a tripod that was placed by ROV on an undisturbed seafloor. A PVC pipe marked with 10 cm divisions was laid on the seabed to provide additional scaling in the field of view. The camera was connected to the interface platform (DCF, Figure 1) that hosted additional sensors and the interface to the VENUS network. Images were uploaded to the archive and cross-referenced to other data by time.

The camera was deployed from September 30, 2009 to February 18, 2010 and was re-deployed after servicing for a 2^nd^ observation period collecting from March 08, 2010 to October 18, 2010. Images were acquired three to five times per week (Monday, Wednesday, Friday, and Monday to Friday, respectively). Images from the first time period were generally taken between 16:00 and 17:00 UTC and, from the second time period, between 19:00 and 21:00 UTC. For each observation, the camera was panned in a 360° sweep with overlapping photos (usually 13) to ensure complete coverage. Return-sweep videos of the same area were acquired between January and October 2010 for behavioural observations. The observed areas were 6.2 m^2^ and 4.4 m^2^ during the first and second periods, respectively. The tripod settled in the soft sediments, bringing the camera about 4 cm closer to the seafloor during the second time period compared to the first.

Animals were counted manually using Image J 1.44 (National Institutes of Health, USA; online ref. http://rsbweb.nih.gov/ij/) and two computer screens viewing adjacent images to minimize duplicate counts. The entire community was quantified although three dominant species formed the focus of this study: the shrimp *Spirontocaris sica* (Hippolytidae), the squat lobster *Munida quadrispina* (Galatheidae) and the flatfish *Lyopsetta exilis* (Pleuronectidae). Visual counts were made by only two people to minimize subjectivity. Each investigator processed the whole set of images: one counted the number of individuals per species; the other estimated bacterial mat surface area and squat lobster individual sizes (see below).

For *Munida quadrispina*, size classes were defined following the size and morphological characteristics of the individuals. Recruits occurred on hard structures as settlers with no walking legs evident. Small squat lobsters had identifiable legs but did not exceed ∼2 cm. Large squat lobster displayed hairs on their chelipeds and exceeded 10 cm in size. The remaining squat lobsters were categorized as medium size.

### 3. Visibility

Visibility was also estimated to determine its effect on animal density counts. The factors that influenced visibility were non-uniform lighting, sediment resuspension by fish, and high zooplankton densities. Visibility “ranks” were defined based on the proportion of each field of view that was visible and the average rank for each sweep was subsequently used in the statistical analyses (Table 2).

**Table 2:** Criteria defined to estimate the visibility in the study area.

Rank	Criteria
1	<25% of image visible
2	25%–50% of image visible
3	50%–75% of image visible
4	>75% of image visible

### 4. Bacterial Mat Quantification

We used perspective grids to quantify bacterial mat coverage on the seafloor. The grids were created in the first image of each sweep, where the PVC scaling ruler was visible on the seafloor, and then applied to all images. Image J 1.44 and GIMP 2.6.11 (The GIMP Development Team; online ref. www.gimp.org) were used for scaling and grid construction. Bacterial mat coverage was recorded in a 15-square window of the full grid, located in the lower center of the image where lighting was most uniform and the angle of view was the least oblique. This approach also minimized double counting of areas of photo overlap within a sweep. The lighting was especially critical during times of low bacterial mat coverage.

Using the grid overlain on each image of every sweep, presence/absence of bacterial mat was scored for each visible square. The ‘intensity’, defined as mat ‘whiteness’, of the mat was also recorded, on an increasing scale of 1 to 3. Occasionally poor visibility prevented the viewing of all 15 squares in the grid. To compensate, the number of visible squares, and squares containing mats, were summed and then averaged for each sweep. Percent mat coverage was then calculated as the average mat squares per sweep divided by the average number visible squares per sweep.

### 5. Statistical Analysis

Species abundances were converted to densities of individuals per m^2^. For each of the three dominant species, a linear regression was performed between log-transformed density data and corresponding hourly average dissolved oxygen concentration, followed by an analysis of variance (ANOVA) that tested the significance of the model terms.

Prior to multivariate analyses, a Hellinger transformation was applied to the species density data [74]. This transformation consists of dividing each species’ abundance by the total abundance of species present at the site and then taking the square root of the ratio. After this transformation, Euclidean methods of analysis, such as canonical redundancy analysis (RDA) (described below), preserve the Euclidean distance among observation dates for abundance data. All calculations were performed using R language functions [75]. Because of irregularities in temporal sampling, cross correlations were not applicable for this time series.

The temporal distribution of the benthic community using the three dominant species was quantitatively described using Principal Coordinates of Neighbour Matrices analysis (PCNM; [43]). This method, more commonly used for spatial analysis of ecological studies, allows the detection and quantification of spatial structure over a wide range of spatial scales detectable by the sampling design. We applied this technique to identify temporal structure based on changes in species densities. In this case, the method used time coordinates of the sampling dates to build a matrix of Euclidean distances among the sampling dates. The matrix of Euclidean distances was then truncated at a user-defined threshold to retain only the distance between neighbouring dates. However, the irregular sampling due to the significant gap in observations between the two deployment periods generated irregular principal coordinates. Their interpretation is complicated because each of them may bear structure at several scales [43]. To overcome this problem, nine ‘empty’ observations were added to the time matrix between February 12 and March 8, 2010 reducing the threshold to a distance of 7 days, which corresponds to the greatest distance between two consecutive observation dates [43]. A principal coordinates analysis on the truncated distance matrix was computed and only the PCNM variables with positive Moran’s I values were kept, from which the extra data points (i.e. nine empty observations) were removed. The resulting principal coordinates (PCNM eigenfunctions) were irregular sinusoids describing all the temporal scales that could be observed in the sampling design. The PCNM variables were generated using the PCNM function implemented in the ‘PCNM’ package [75]. The method generated 100 PCNM variables, 49 of which had positive Moran’s I values. These variables were then used in a canonical redundancy analysis (RDA), implemented in the ‘vegan’ package [75], to explain the variation in community structure (response variables) among observations dates. The RDA combines aspects of ordination and regression and uses a permutation procedure to test the significance of the explained variation; thus species density data do not have to be normally distributed [74].

A forward selection procedure identified 26 significant PCNM functions [76] implemented in the ‘packfor’ package [75]. This procedure uses the results of a permutation test (999 random permutations) to test the significance of the explanatory variables (PCNM functions) successively entering the model. It stops when either the p-value of a newly included variable is higher than an alpha threshold of 0.05, or the contribution (adjusted R^2^) of a newly included variable is lower than a threshold equal to the adjusted R^2^ value from the initial RDA. The 26 PCNM functions were grouped visually according to their respective sinusoid periods, leading to the identification of three scales in the temporal variability of abundances. To estimate the time period for each PCNM grouping, sub-models were created by combining the PCNM variables within each grouping. RDAs were used to test the significance of each sub-model (i.e. time scale) on the biological matrix, followed by canonical analyses of variance to test the significance of each RDA axis. The significant axes were fitted in a linear model to identify the significant contribution of environmental variables in each sub-model.

A forward selection procedure, followed by a canonical redundancy analysis (RDA) was also conducted on the entire biological matrix to determine the role of environmental variables in shaping the entire benthic community structure. The significance of the global RDA and of each axis was tested using a canonical analysis of variance.
